# Total serum cholinesterase activity predicts hemodynamic changes during exercise and associates with cardiac troponin detection in a sex-dependent manner

**DOI:** 10.1186/s10020-018-0063-0

**Published:** 2018-12-18

**Authors:** Rafael Y. Brzezinski, Eyal Fisher, Noa Cohen, Etti Zwang, Gabi Shefer, Naftali Stern, David Zeltser, Itzhak Shapira, Shlomo Berliner, Ori Rogowski, Shani Shenhar-Tsarfaty

**Affiliations:** 1 Department of Internal Medicine “C”, “D” and “E”, Tel Aviv Sourasky Medical Center and Sackler Faculty of Medicine, Tel Aviv University, 6 Weizmann Street, Tel Aviv, 64239 Israel; 2 Sackler Faculty of Medicine, Tel Aviv University, Tel Aviv, Israel; 3 Department of Applied Mathematics and Theoretical Physics, University of Cambridge, Cambridge, UK; 4 Institute of Endocrinology, Metabolism and Hypertension, Tel Aviv Sourasky Medical Center, Tel Aviv University, Tel Aviv, Israel

**Keywords:** Autonomic nervous system, Parasympathetic dysfunction, Exercise stress test, Sex difference, Cardiac troponin, Cholinesterase

## Abstract

**Background:**

Imbalanced autonomic nervous system (ANS) activity is associated with poor cardiovascular outcome. However, clinically validated biomarkers to assess parasympathetic function are not yet available. We sought to evaluate parasympathetic dysfunction by measuring serum cholinesterase activity and to determine its relationship to high sensitive cardiac troponin T (hs-cTnT) as well as traditional non-invasive parameters of ANS function during exercise in apparently healthy individuals.

**Methods:**

We enrolled 1526 individuals (mean age 49 ± 11 yr., 75% men) from the Tel Aviv Medical Center Inflammation Survey (TAMCIS). We used the acetylcholine (ACh) analog acetylthiocholine (ATCh) as a substrate that is hydrolyzed by both ACh degrading enzymes and reflects the total serum capacity for acetylcholine hydrolysis, referred to as cholinergic status (CS). All subjects performed a cardiac stress test reviewed on the spot by a cardiologist and multiple physiological and metabolic parameters including hs-cTnT were measured.

**Results:**

CS values at rest predicted multiple exercise-hemodynamic changes. Heart rate recovery after exercise was inversely correlated to CS values (*p* < 0.01 and *p* = 0.03 for women and men respectively), and a hypertensive reaction during exercise was associated with elevated CS levels in women. Most importantly, women with detectable hs-cTnT (> 5 ng/L) presented with elevated CS levels compared to women with undetectable levels; 1423 ± 272.5 vs 1347 ± 297.9 (*p* = 0.02). An opposite trend was observed in men. Metabolic dysfunction parameters were also associated with CS elevation in both men and women.

**Conclusions:**

Parasympathetic dysfunction assessed by total serum cholinesterase activity predicts hemodynamic changes during exercise. CS is also associated with hs-cTnT detection in women and inversely so in men. Future studies to assess the potential clinical use of this new sex-specific biomarker in cardiovascular disease risk stratification are warranted.

**Graphical Abstract:**

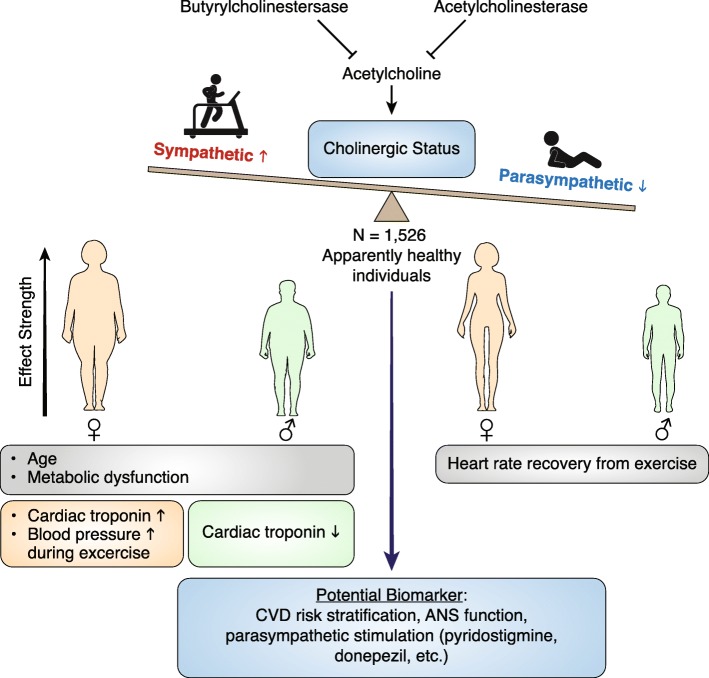

**Electronic supplementary material:**

The online version of this article (10.1186/s10020-018-0063-0) contains supplementary material, which is available to authorized users.

## Background

Imbalanced autonomic nervous system (ANS) activity is associated with poor cardiovascular outcome (Lahiri et al. 2008). More specifically, decreased parasympathetic activity has been related to metabolic impairment, systemic inflammation and future major adverse cardiovascular events (MACE) (Shenhar-Tsarfaty et al. 2014; Rao et al. 2007; Hansen et al. 2017). However, clinically validated biomarkers to assess the parasympathetic system are not yet available and the main neurotransmitter-acetylcholine (ACh) is extremely labile and difficult to measure in the circulation. Therefore, the use of its hydrolyzing enzymes as an indirect measurement for parasympathetic dysfunction could serve as a surrogate marker in many clinical settings (Shenhar-Tsarfaty et al. 2014). Our method uses the ACh analog acetylthiocholine (ATCh) as a substrate that is hydrolyzed by both ACh degrading enzymes (acetylcholinesterase and butyrylcholinestersase) and reflects the total serum capacity for ACh hydrolysis, referred to as cholinergic status (CS).

Traditional non-invasive parameters of ANS dysfunction e.g. heart rate and blood pressure profile during exercise are predictors of worse cardiovascular prognosis (Lahiri et al. 2008; Jouven et al. 2005). Specifically, an exaggerated blood pressure response to exercise is associated with a greater risk of developing hypertension and increased prevalence of left ventricular hypertrophy in otherwise normotensive adults (Kim and Ha 2016). However, indications to start treatment based on these parameters, and the prognostic value of such interventions are inconclusive (Kim and Ha 2016; Chant et al. 2018).

High sensitive cardiac troponin T (hs-cTnT) could potentially aid in cardiovascular disease (CVD) risk stratification among individuals presenting with abnormal heart rate and blood pressure profile during exercise. hs-cTnT is related to future cardiovascular events in the general population and in patients with ischemic heart disease, with recent reports demonstrating evident structural changes in CVD-free adults with elevated concentrations (Willeit et al. 2017; Seliger et al. 2017). Of note, recent reports highlighting sex- and gender-related differences in circulating biomarkers of CVD call for a more gender-oriented clinical and research approach (Daniels and Maisel 2015).

Therefore, we sought to examine the relationship between total serum cholinesterase activity and hs-cTnT alongside traditional hemodynamic parameters of ANS function during exercise. We aimed to evaluate our novel biomarker in a cohort of apparently healthy individuals and to determine sex- and gender-related differences.

## Methods

### Study population

We enrolled 1526 individuals (mean age 49 ± 11 yr., 75% men) from the Tel Aviv Medical Center Inflammation Survey (TAMCIS). TAMCIS encompasses a large cohort of apparently healthy subjects who attend the Tel Aviv Medical Center for routine annual checkups. These include a physician’s interview and examination, blood and urine tests, and a cardiac exercise ECG stress test (EST). Participants were enrolled consecutively between February 2016 and June 2017. Patients between the age of 18–90 were included in the study. Participants’ characteristics as well as information on cardiovascular co-morbidities are shown in Table 1. The study was approved by the local ethics committee, conformed to the principles outlined in the Declaration of Helsinki, and informed consent was obtained from all participants as detailed in previous reports (Brzezinski et al. 2018; Brzezinski et al. 2017).

**Table 1:** Population Characteristics by Sex

Characteristic	Men	Women	p - value
N	1153 (75%)	373 (25%)	
Age, years	50 (11)	47 (12)	0.001
BMI, Kg/m^2^	27 (4)	25 (5)	< 0.001
Resting Pulse, beats/min	69 (14)	74 (11)	0.002
Systolic BP, mmHg	130 (16)	119 (20)	< 0.001
Diastolic BP, mmHg	80 (34)	73 (12)	0.001
Peak systolic BP, mmHg	173 (23)	156 (23)	< 0.001
Peak diastolic BP, mmHg	72 (13)	70 (13)	0.091
METs	12 (2)	10 (6)	< 0.001
Cholinergic status, normalized nmol/min/ml	1471 (342)	1370 (291)	< 0.001
hs-cTnT, ng/L	5 [3^a^,7]	3.0 [3^a^,4]	< 0.001
Fasting glucose, mg/dl	89 (14)	84 (9)	< 0.001
HbA1c, %	5.4 [5.2, 5.7]	5.4 [5.1, 5.6]	0.054
T-C, mg/dl	186 (33)	192 (33)	0.004
HDL-C, mg/dl	47 (12)	61 (15)	< 0.001
LDL-C, mg/dl	115 (28)	111 (28)	0.032
TG, mg/dl	121 (65)	100 (49)	< 0.001
eGFR, mL/min/1.73m^2^	94 (14)	100 (15)	< 0.001
UACR, mg/g	2 [1, 4]	3 [2, 7]	< 0.001
hs-CRP, mg/l	2 (2.9)	2.7 (3.7)	< 0.001
Diabetes diagnosis (%)	62 (7)	8 (3)	0.016
Hypertension (%)	182 (17)	32 (9)	< 0.001
IHD diagnosis (%)	53 (6)	13 (5)	0.470
Use of antihypertensive- medication (%)	134 (12)	17 (5)	< 0.001
Hyperlipidemia (%)	97 (9)	25 (7)	0.307

*BP* blood pressure, *BMI* body mass index, *METs* Metabolic equivalents; *hs-cTnT* high sensitive cardiac troponin T, *HbA1c* Hemoglobin A1C, *T-C* total cholesterol, *HDL-C* high density lipoprotein cholesterol, *LDL-C* low density lipoprotein cholesterol, *TG* triglycerides, *eGFR* estimated glomerular filtration rate, *UACR* urinary albumin/creatinine ratio, *hs-CRP* high sensitive C- reactive protein, *IHD* ischemic heart disease. Values are mean (SD) or median [interquartile range]

^a^ Equivalent to non-detectable hs-cTnT (below detection level of 5 ng/L), as described in the Methods section

We collected blood samples for the measurement of multiple physiologic and metabolic parameters including hs-cTnT, lipid profile, glucose, hemoglobin A1c (HbA1C) and CS levels. Blood samples were obtained at rest before the participants performed the EST.

### Cholinergic status measurement

Sympathetic-parasympathetic balance was assessed as previously described by our group (Shenhar-Tsarfaty et al. 2014). We used the ACh analog acetylthiocholine (ATCh) as a substrate that is hydrolyzed by both ACh degrading enzymes (acetylcholinesterase and butyrylcholinestersase) and reflects the total serum capacity for acetylcholine hydrolysis, referred to as CS.

Serum samples were frozen at − 80 °C until acetylcholine hydrolysis analysis. Acetylcholinesterase and total cholinesterase activity levels were assayed in triplicates in a microtiter plate using an adaptation of the Ellman assay (ELLMAN et al. 1961). Hydrolysis of 1 mM acetylthiocholine (ATCh, Sigma) was followed by spectrofluorometry (Spectrafluor Plus, Tecan) at 405 nm. Prior to read, we incubated the samples for 20 min in the dark with (for acetylcholinesterase activity) or without (for total cholinesterase activity) 50 μM tetra isopropyl pyrophosphoramide (iso-OMPA, Sigma) which is a specific butyrylcholinestersase inhibitor.

We calculated enzyme activity using 13,600 M/cm as the e405 for 5-thio-2-nitrobenzoate. Total cholinergic activity is termed CS (Shenhar-Tsarfaty et al. 2015). The calculated coefficient of variation varies from 3 to 13% depending on the tested cohort (minimal with control, varying with acute phase patients such as those with myocardial infarction or stroke); the intra assay is 6.75%, enabling good discrimination between the groups.

### Exercise-hemodynamic parameters

All subjects performed an EST according to the Bruce protocol. The test was operated by a trained technician and the results were analyzed on the spot by a cardiologist.

A hypertensive reaction during exercise was considered as values above the 90th percentile cutoff of peak systolic blood pressure (SBP) values; > 205 mm/Hg in men and > 187 mm/Hg in women. These values are similar to previous study populations (Kim and Ha 2016; Lewis et al. 2008). SBP elevation rate was calculated as peak SBP minus SBP at rest divided by time (in min) to peak. Heart rate recovery rate was calculated as absolute values of heart rate upon recovery minus peak rate divided by the recovery time (min). Men and women were divided into sex-specific quartiles, starting with individuals with low heart rate recovery rate at the bottom quartiles and ones with fast recovery rates at the top quartiles.

### Measurement of high-sensitive cardiac troponin T and other metabolic parameters

hs-cTnT was measured using a sandwich immunoassay method with a Roche Elecsys 2010 Analyser (Roche Diagnostics). The range of the assay is 3–100,000 ng/L with levels below limit of blank (levels that are not measurable and are recorded as blank) of 3 ng/L. (Agarwal et al. 2011) Of note, hs-cTnT levels between 3 and 5 ng/L are measurable but with lower precision than concentrations ≥5 ng/L. (Saunders et al. 2011; McEvoy et al. 2016) Thus, 5 ng/L is the limit of detection of this assay and was used as the cut-off for our detectable hs-cTnT category. Levels of 14 ng/L or greater represented the 97th percentile in the TAMCIS study sample and the 99th percentile value for a healthy reference group aged 20–70 years (Giannitsis et al. 2010).

We separated A1c from non-glycated hemoglobin of whole blood samples in EDTA with Tosoh’s G7 HPLC (Tosoh Bioscience, Inc. San Francisco, CA, USA). HbA1c levels were categorized as: healthy- < 5.7%; pre-diabetic - 5.7–6.4%; and diabetic - > 6.5%, according to the American Diabetes Association guidelines (Care and Suppl 2018)..

Evaluation and diagnosis of metabolic syndrome (and its components) were performed based on the joint interim statement of the International Diabetes Federation Task Force on Epidemiology and Prevention; National Heart, Lung, and Blood Institute; American Heart Association; World Heart Federation; International Atherosclerosis Society; and International Association for the Study of Obesity (Alberti et al. n.d.). Briefly, elevated waist circumference was defined as ≥102 cm in men and ≥ 80 cm in women, as recommended for individuals of European and Middle Eastern descent. Elevated triglycerides were defined as ≥150 mg/dl (1.7 mmol/l) or on drug treatment for elevated triglycerides. Reduced high-density lipoprotein-cholesterol (HDL) was defined as < 40 mg/dl (1.0 mmol/l) in men and < 50 mg/dl (1.3 mmol/l) in women. Elevated blood pressure was defined as ≥130 mmHg for SBP or ≥ 85 mmHg for diastolic blood pressure, or on antihypertensive drug treatment in a patient with a history of hypertension. Elevated fasting glucose was defined as ≥100 mg/dl (5.55 mmol/l). The diagnosis of metabolic syndrome was based on the existence of at least three abnormal findings out of the five mentioned above.

### Statistical analysis

All continuous variables are displayed as means (SD) for normally distributed variables or median [interquartile range] for variables with abnormal distribution. Categorical variables are displayed as numbers (%) of subjects within each group. The different biomarkers in men and women were compared by a Student’s *t* test for normally distributed variables and by the Mann-Whitney *U* test for non-normally distributed ones. To assess associations among categorical variables, we used a chi-square test.

We performed a one-way Analysis of Variance (ANOVA) with a linear contrast to compare the CS values between sex-specific quartiles of heart recovery. A Student’s t test was used to compare CS values between the different binary variables, i.e. subjects with a normal vs hypertensive reaction during exercise, healthy vs. pre-diabetic individuals and subjects with detectable vs. non-detectable hs-cTnT.

In order to identify possible confounders, we performed a multivariate regression aimed to predict a hypertensive reaction during exercise. The model was controlled for the following covariates: categorized sex-specific quartiles of CS, age, body mass index (BMI), systolic and diastolic blood pressure at rest and metabolic equivalents (METs). Pearson’s test was used to assess the correlation between CS values and SBP elevation rate. Spearman’s test was used to assess the correlation between CS values and hs-cTnT concentrations.

A one-way ANOVA was used to evaluate the difference in BMI and the number of metabolic syndrome components between the different sex-specific quartiles of CS values. A multivariate regression was used to predict detectable hs-cTnT status (> 5 ng/L) with the following covariates: age (in years), number of metabolic syndrome components and sex-specific quartiles of CS values.

*P* values of < 0.05 were considered statistically significant. We used the R statistical package (version 3.3.1, R Foundation for Statistical Computing, Vienna, Austria) along with IBM SPSS Statistics 22.0 statistical package (IBM Corporation, Armonk, New York, USA) and GraphPad Prism version 7.00 (GraphPad Software, La Jolla, CA, USA) for all statistical analysis.

## Results

Participants’ characteristics are shown in Table 1. Men had a higher prevalence of hypertension and diabetes along with slightly higher BMI, glucose and blood lipids levels. Among 1526 participants, 144 demonstrated a hypertensive reaction during exercise (~ 9% for both men and women).

### CS at rest predicts hemodynamic parameters during exercise

Concordant with previous reports, overall CS values were lower in women compared to men (Table 1) (Arbel et al. 2014).

**Fig. 1:**
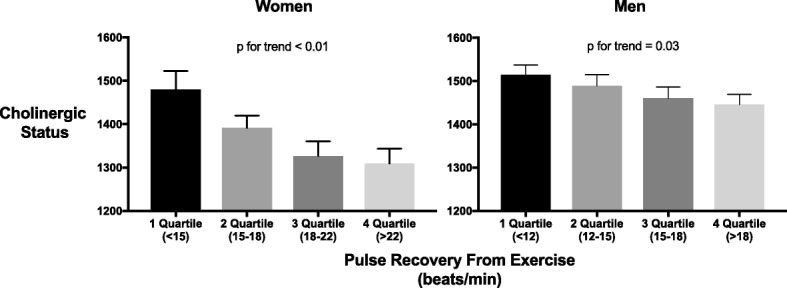
CS values decrease between sex-specific quartiles of heart rate recovery from exercise. Women (left) and Men (right) were divided into quartiles according to pulse recovery from exercise rate (beats/min). CS values represent total serum rates of ATCh hydrolysis (substrate hydrolyzed per minute per milliliter). CS values were inversely correlated to heart rate recovery from exercise in both women and men (p for trend < 0.01, = 0.03 respectively). Presented are mean ± SEM. p by one-way ANOVA with a linear contrast for trend analysis. *ATCh –* Acetylthiocholine; *CS* - Cholinergic status

**Fig. 2:**
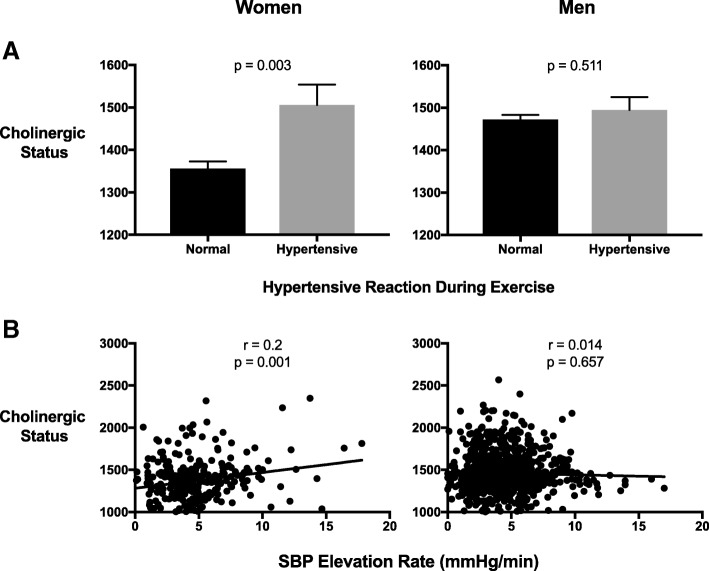
Cholinergic Status correlates with blood pressure elevation during exercise in women but not in men. **a** CS values are higher in women with a hypertensive reaction during exercise (defined as SBP > 187 mm/Hg in women (left) and > 205 mm/Hg in men (right)). **b** CS values in women correlate with SBP elevation rate (*r* = 0.2, *p* = 0.001) calculated as peak SBP minus SBP at rest divided by time to peak. CS values represent total serum rates of ATCh hydrolysis (substrate hydrolyzed per minute per milliliter). Presented are mean ± SEM. p by student’s T-test (**a**) and Pearson’s correlation test (**b**). *SBP* - Systolic blood pressure; *ATCh -* Acetylthiocholine; *CS* – Cholinergic status

CS values at rest predicted multiple exercise-hemodynamic changes in a sex-dependent manner, demonstrating significant trends in women rather than men. In women, heart rate recovery after exercise was inversely correlated to CS values, with decreasing levels evident between heart rate recovery quartiles (Fig. 1). Furthermore, a hypertensive reaction during exercise was associated with elevated CS values; 1506 ± 277.1 for mean ± SD in hypertensives vs. 1356 ± 290.1 in normotensive women (*p* = 0.003, Fig. 2). This finding remained significant after controlling for age, BMI, systolic and diastolic blood pressure at rest and METs (Table 2).

**Table 2:** Multivariate regression to predict a hypertensive reaction during exercise in women and men

Covariates	Women				Men			
	OR	p	95% CI- Lower limit	95% CI- Upper limit	OR	p	95% CI- Lower limit	95% CI- Upper limit
CS (1st quartile)		.221				.474		
CS (2nd quartile)	3.717	.145	.635	21.762	.976	.942	.510	1.868
CS (3rd quartile)	4.701	.090	.785	28.142	1.492	.210	.798	2.791
CS (4th quartile)	6.301	.037	1.116	35.575	1.282	.438	.684	2.402
Age (years)	1.043	.058	.999	1.090	.998	.871	.977	1.020
BMI	1.083	.095	.986	1.188	1.102	.001	1.041	1.166
Systolic BP at rest	1.065	.000	1.031	1.099	1.068	.000	1.050	1.086
Diastolic BP at rest	1.011	.646	.965	1.058	.988	.317	.966	1.011
METs	.956	.705	.759	1.205	1.065	.218	.964	1.177

*OR* odds ratio, *CI* confidence intervals, *CS* cholinergic status, *BMI* body mass index, *BP* blood pressure, *METs* Metabolic equivalents

CS was also correlated with the SBP elevation rate; *r* = 0.2, *p* = 0.001, suggesting this biomarker reflects upon arterial vasoconstriction in normal and hypertensive adult women (Fig. 2).

### CS elevation is associated with metabolic impairment

Next, we sought to examine whether elevated CS values also correlate with metabolic impairment. HbA1c levels in women were significantly correlated with CS values; *r* = 0.18, *p* < 0.001. Also, pre-diabetic women (HbA1C > 5.7%) presented higher CS values than healthy ones; 1447.8 ± 296.8 vs. 1355.58 ± 289.711 for mean ± SD respectively (*p* = 0.03).

Trend analysis revealed elevated BMI and accumulating components of the metabolic syndrome across sex-specific quartiles of CS in both men (p for trend = 0.019, 0.046) and women (p for trend = 0.018, < 0.001).

### CS is positively associated with hs-cTnT detection in women and inversely so in men

**Fig. 3:**
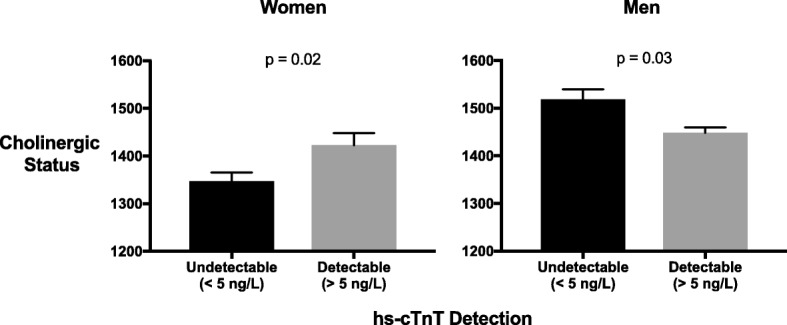
CS values are higher in women with detectable hs-cTnT (> 5 ng/L) than in women with undetectable levels. CS values represent total serum rates of ATCh hydrolysis (substrate hydrolyzed per minute per milliliter). An opposite trend was observed in men. Presented are mean ± SEM. p by Student’s T- test. *hs-cTnT* - High sensitive cardiac troponin T; *ATCh* – Acetylthiocholine; *CS* – Cholinergic status

Finally, and most importantly, we aimed to evaluate sub-clinical myocardial damage through the use of circulating hs-cTnT. Interestingly, opposite trends were observed between the sexes. Detectable hs-cTnT in women was correlated with elevated CS levels; 1423 ± 272.5 for mean ± SD in women with detectable hs-cTnT (> 5 ng/L) vs 1347 ± 297.9 in undetectable subjects (*p* = 0.02, Fig. 3). In contrast, men with detectable circulating cardiac troponin presented lower levels of CS compared with men with undetectable levels (p = 0.03, Fig. 3). CS and hs-cTnT demonstrated a weak yet significant linear correlation. This correlation was also sex specific; positive in women (*r* = 0.1, *p* = 0.047) and negative in men (*r* = − 0.1, *p* = 0.001).

**Fig. 4:**
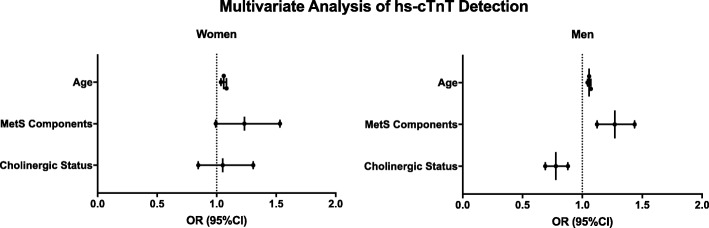
Sex difference in CS prediction of hs-cTnT detection. We performed a multivariate logistic regression to predict detectable hs-cTnT status (> 5 ng/L) with the following covariates: age (in years), number of metabolic syndrome components and sex-specific quartiles of CS values. CS was correlated with hs-cTnT detection in women, albeit not statistically significant (OR = 1.05, *p* = 0.6). This correlation is inversed in men (OR = 0.8, *p* < 0.001). Presented are odds ratio (OR) with 95% confidence intervals (CI). *hs-cTnT* - High sensitive cardiac troponin T; *MetS* - Metabolic syndrome; *CS* – Cholinergic status

Multivariate analysis adjusted for age and metabolic profile (defined as number of metabolic syndrome components), revealed that elevated CS values predicted undetectable hs-cTnT status in men compared to detectable status in women, albeit not statistically significant in women (Fig. 4). Age was identified as a significant predictor in the model for both sexes. Of note, after controlling for age, CS remained a significant predictor for hs-cTnT detection in men but not in women (Fig. 4, Additional file 1: Table S1). CS and hs-cTnT elevation might be associated with the aging female. The number of metabolic syndrome components was associated with hs-cTnT detection in both sexes (Fig. 4, Additional file 1: Table S1), similar to previous findings (Milwidsky et al. 2014; Pokharel et al. 2017).

## Discussion

**Fig. 5:**
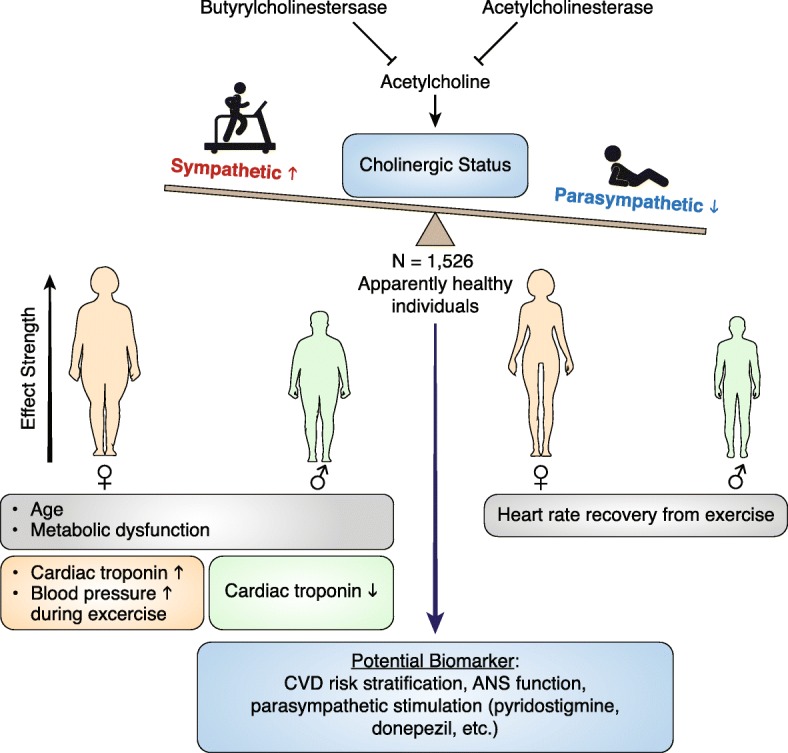
Summary Figure.The acetylcholine analog acetylthiocholine is hydrolyzed by two degrading enzymes (acetylcholinesterase and butyrylcholinestersase). Its degredation reflects the total serum cholinesterase activity referred to as Cholinergic Status (CS). CS potentially reflects upon the body’s sympathetic-parasympathetic balance with decreased levels of CS indicating prominent parasympathetic activity. In both sexes, CS was correlated with age and metabolic impairment. In women, elevated CS values predicted a hypertensive reaction during exercise and correlated with systolic blood pressure elevation rate. Most importantly, CS is positively correlated with high sensitive cardiac troponin T detection in women but inversely so in men. Heart rate recovery from exercise was inversely correlated to CS in both sexes, more significantly in women. CS is a potential new biomarker for cardiovascular disease risk stratification, autonomic nervous system function and monitoring of parasympathetic stimulation treatment strategies. *ANS- Autonomic Nervous System*

The main finding of this study is the positive association between serum cholinesterase activity (CS values) and hs-cTnT detection in women rather than men (Fig. 5). To our knowledge, this marks the first attempt to describe the interaction between ANS dysfunction and hs-cTnT elevation in apparently healthy individuals.

Sex-related differences in circulatory biomarkers for CVD risk stratification are becoming increasingly relevant. hs-cTnT levels are generally lower in women compared to men, at both baseline and in the setting of acute coronary syndrome (Daniels and Maisel 2015). The exact reason remains elusive but might relate to the smaller cardiac mass in females. The positive association between CS and hs-cTnT detection seen in women rather than men call for future mechanistic understanding of ANS dysfunction and myocyte injury in the female heart. Decreased parasympathetic activity, measured here as elevated values of CS, is also associated with a systemic pro-inflammatory state (Shenhar-Tsarfaty et al. 2014; Shenhar-Tsarfaty et al. 2016), suggesting a possible cause for cardiac necrosis and structural changes evident as detectable hs-cTnT levels. We hypothesize that low-grade systemic inflammation leads to more elevated levels of CS in women rather than men, possibly explaining the sex-related difference seen in the association between hs-cTnT detection and CS values. Our findings, along with previous well-established reports on hs-cTnT elevation and future risk for MACE (Willeit et al. 2017; Seliger et al. 2017), place this novel biomarker in a significant clinical perspective, especially among women.

In women, the association between CS and hs-cTnT detection was not statistically significant after controlling for age. However, CS remained inversely correlated with hs-cTnT in men. It is possible that menopausal status is involved in the regulation of CS and hs-cTnT and is thus responsible for this sex-related difference. Future studies aimed at assessing the influence of sex hormones on CS throughout women’s life-span are needed.

Our sex-specific thresholds for CS values and hemodynamic parameters of exercise promote the idea of implying more sex-specific thresholds for biomarkers in both research and clinical settings of CVD. Future development of the high sensitivity assay for cardiac troponin will enable these specific thresholds also for apparently healthy individuals who present with relatively low circulatory concentrations.

The association between CS values and hemodynamic changes during an EST contributes additional insight into the multifactorial etiology of exaggerated blood pressure response during exercise (Kim and Ha 2016). Serum cholinesterase activity seems to be an important contributor, placing ANS dysfunction as a potential therapeutic target to treat hypertension.

Recent reports have demonstrated that pharmaceutical parasympathetic stimulation with cholinesterase inhibitors (e.g. pyridostigmine, donepezil, etc.) is effective in both left and right heart failure, alongside associated pulmonary and systemic arterial hypertension in experimental models and humans (Okazaki et al. 2010; da Silva Gonçalves Bos et al. 2017). We present here a relatively accurate and clinically-applicable method to monitor these new therapeutic strategies, potentially helping to integrate them into future clinical practice. Future studies on the effects of these interventions on CS are warranted.

Our study is limited by the lack of follow-up data on the prevalence of MACE in our cohort, thus limiting the prognostic evaluation of this new biomarker. Future larger studies with adequate follow-up periods are needed in order to determine whether CS measurements can provide additional prognostic value to existing biomarkers of CVD, in either healthy adults or in individuals with existing co-morbidities. Furthermore, the observational nature of our findings limits our ability to determine causality between CS and hs-cTnT elevation. The significant trends presented here in a relatively large sample of patients call for future experimental studies to determine the underlying mechanism for changes in CS levels in both males and females. Of note, our study population consists largely of male participants (75%), thus potentially affecting data interpretation. However, the female group still consists of a relatively large number of observations (*n* = 373) and all statistical analysis was performed separately in men and women with the use of sex-specific thresholds.

## Conclusions

We conclude that CS is able to predict multiple hemodynamic changes during exercise, especially in women. CS values demonstrate opposite trends between men and women regarding its association with the detection of circulating cardiac troponin. CS is a potential biomarker for decreased parasympathetic activity and ANS dysfunction. Its relatively rapid, accurate and applicable nature, calls for further investigation of its use in clinical settings of CVD risk stratification in the general population, especially in women.

## Additional file

Additional file 1: **Table S1.** Multivariate Regression to Predict hs-cTnT Detection Status (> 5 ng/L) in Men and Women. (DOCX 19 kb)

## Abbreviations

ACh: Acetylcholine
ANS: Autonomic nervous system
ATCh: Acetylthiocholine
BMI: Body mass index
CVD: Cardiovascular disease
EST: Exercise ECG stress test
HbA1c: Hemoglobin A1c
hs-cTnT: High sensitive cardiac troponin T
MACE: Major adverse cardiovascular events
METs: Metabolic equivalents
SBP: Systolic blood pressure
