# Supplementary material for: Total serum cholinesterase activity predicts hemodynamic changes during exercise and associates with cardiac troponin detection in a sex-dependent manner
Source: Mol Med. 2018 Dec 18;24:63. doi: 10.1186/s10020-018-0063-0 (PMC6299630; doi:10.1186/s10020-018-0063-0)
Supplement: Supplementary file 1 — Table S1. Multivariate Regression to Predict hs-cTnT Detection Status (> 5 ng/L) in Men and Women. (DOCX 19 kb) [file 10020_2018_63_MOESM1_ESM.docx]

Table S1: **Multivariate Regression to Predict hs-cTnT Detection Status (>5 ng/L) in Men and Women**

1. **Men**

|  | **Model 1** | | **Model 2** | | **Model 3** | |
| --- | --- | --- | --- | --- | --- | --- |
|  | OR (95% CI) | p | OR (95% CI) | p | OR (95% CI) | p |
| **CS (1^st^ quartile)** |  | .002 |  | .001 |  | .001 |
| **CS (2^nd^ quartile)** | .93 (.64-1.36) | .739 | .94 (.63-1.39) | .770 | .92 (.62-1.37) | .708 |
| **CS (3^rd^ quartile)** | .75 (.51-1.08) | .127 | .63 (.43-.93) | .021 | .61 (.41-.90) | .013 |
| **CS (4^th^ quartile)** | .53 (.37-.76) | .001 | .52 (.36-.76) | .001 | .50 (.34-.73) | .000 |
| **Age (years)** |  |  | 1.06 (1.04-1.07) | .000 | 1.05 (1.04-1.07) | .000 |
| **MetS Components**  **(Count)** |  |  |  |  | 1.27 (1.12-1.43) | .000 |

1. **Women**

|  | **Model 1** | | **Model 2** | | **Model 3** | |
| --- | --- | --- | --- | --- | --- | --- |
|  | OR (95% CI) | p | OR (95% CI) | p | OR (95% CI) | p |
| **CS (1^st^ quartile)** |  | 0.296 |  | .683 |  | .682 |
| **CS (2^nd^ quartile)** | 1.58 (.82-3.05) | 0.171 | 1.48 (.74-2.96) | .264 | 1.54 (.77-3.09) | .221 |
| **CS (3^rd^ quartile)** | 1.63 (.84-3.15) | 0.144 | 1.39 (.695-2.787) | .351 | 1.30 (.64-2.62) | .464 |
| **CS (4^th^ quartile)** | 1.83 (.95-3.51 | 0.067 | 1.41 (.71-2.80) | .325 | 1.27 (.63-2.56) | .495 |
| **Age (years)** |  |  | 1.06 (1.04-1.08) | .000 | 1.06 (1.03-1.08) | .000 |
| **MetS Components**  **(Count)** |  |  |  |  | 1.25 (1.00-1.55) | .044 |

*OR - Odds ratio; CI - Confidence intervals; CS - Cholinergic status; Mets – Metabolic Syndrome*
